# Supplementary material for: Synthesis of MnO/C/NiO-Doped Porous Multiphasic Composites for Lithium-Ion Batteries by Biomineralized Mn Oxides from Engineered Pseudomonas putida Cells
Source: Nanomaterials (Basel). 2021 Feb 1;11(2):361. doi: 10.3390/nano11020361 (PMC7912735; doi:10.3390/nano11020361)
Supplement: Supplementary file 1 [file nanomaterials-11-00361-s001.pdf]

Supplementary Material: Synthesis of MnO/C/NiO-Doped Porous Multiphasic Composites for Lithium-Ion Batteries by Biomineralized Mn Oxides from Engineered *Pseudomonas putida* Cells

Jin Liu, Tong Gu, Li Li and Lin Li *

Supplementary Tables

Table S1. Compositions of surface elements in biogenic MnO_2/bacteria composites analyzed by line-scan EDS.

Elements	Wt (%)	Atomic (%)
N	19.28	23.62
O	63.99	68.71
Na	2.73	2.04
Mg	0.21	0.07
P	0.2	2.12
K	0.78	0.34
Ca	1.53	0.66
Mn	7.78	2.43

Table S2. Near-surface composition of Mn species derived from fittings of Mn ($2p_{3/2}$) spectra.

Peak	B.E (eV)	FWHM (eV)	Percent (%) BMB	B800	CMB- Ni	Surface species & Comments
**Mn^{2+} ($2p_{3/2}$) parameters**			Mn^{2+} (total) = 30.25 ± 0.1 At. %	Mn^{2+} (total) = 81.44 ± 0.3 At. %	Mn^{2+} (total)= 75.22 ± 0.3 At. %	
Mn^{2+}	639.75	1.25	8.5	11.83	11.62	**Mn(Ⅱ)-O Multiplet #1**
Mn^{2+}	640.95	1.25	18.75	19.33	21.93	**Mn(Ⅱ)-O Multiplet #2**
Mn^{2+}	641.75	1.25	0.5	19.72	12.94	**Mn(Ⅱ)-O Multiplet #3**
Mn^{2+}	642.65	1.25	0	16.56	16.67	**Mn(Ⅱ)-O Multiplet #4**
Mn^{2+}	644.15	1.25	0	10.06	9.65	**Mn(Ⅱ)-O Multiplet #5**
Mn^{2+}	646.4	1.25	2.5	3.94	2.41	**Mn(Ⅱ)-O Multiplet #6**
**Mn^{3+} ($2p_{3/2}$) parameters**			Mn^{3+} (total) = 6 ± 0.3 At. %	Mn^{3+} (total) = 9.66 ± 0.1 At. %	Mn^{3+} (total) = 12.06 ± 0.1 At. %	
Mn^{3+}	640.65	1.25	4.25	0.2	0.22	**Mn(Ⅲ)-O Multiplet #1**
Mn^{3+}	641.35	1.25	0	0.59	0.22	**Mn(Ⅲ)-O Multiplet #2**
Mn^{3+}	642.16	1.25	0.5	0.39	2.63	**Mn(Ⅲ)-O Multiplet #3**
Mn^{3+}	643.18	1.25	0.5	8.28	7.24	**Mn(Ⅲ)-O Multiplet #4**
Mn^{3+}	644.55	1.25	0.75	0.2	1.75	**Mn(Ⅲ)-O Multiplet #5**
**Mn^{4+} ($2p_{3/2}$) parameters**			Mn^{4+} (total) = 63.75 ± 0.3 At. %	Mn^{4+} (total) = 8.9 ± 0.1 At. %	Mn^{4+} (total) = 12.72 ± 0.1 At. %	
Mn^{4+}	641.90	1.25	25	0.2	4.17	**Mn(Ⅳ)-O Multiplet #1**
Mn^{4+}	642.92	1.25	19.5	0.41	0.22	**Mn(Ⅳ)-O Multiplet #2**
Mn^{4+}	643.75	1.25	10	0.2	1.97	**Mn(Ⅳ)-O Multiplet #3**
Mn^{4+}	644.78	1.25	6.25	4.34	2.85	**Mn(Ⅳ)-O Multiplet #4**
Mn^{4+}	645.80	1.25	3	3.75	3.51	**Mn(Ⅳ)-O Multiplet #5**

Note: [a] Abbreviations: BE, binding energy; FWHM, full width at half maximum; At., atoms.

Table S3. Composition of surface elements in B800 analyzed by line-scan EDS.

Elements	Wt %	Atomic %
C	9.78	18.52
O	32.20	45.76
F	2.51	3
Na	5.88	5.82
Mg	1.27	1.19
P	15.77	11.58
S	0.32	0.23
K	0.55	0.32
Ca	2.96	1.68
Mn	28.75	11.90

Supplementary Figures

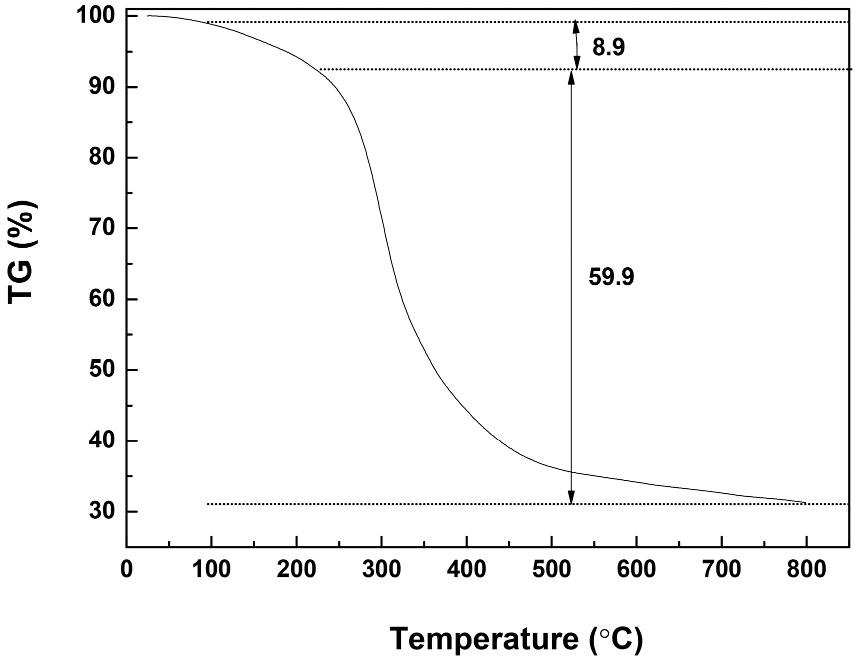

Figure S1. TGA-DSC curves of BMB.

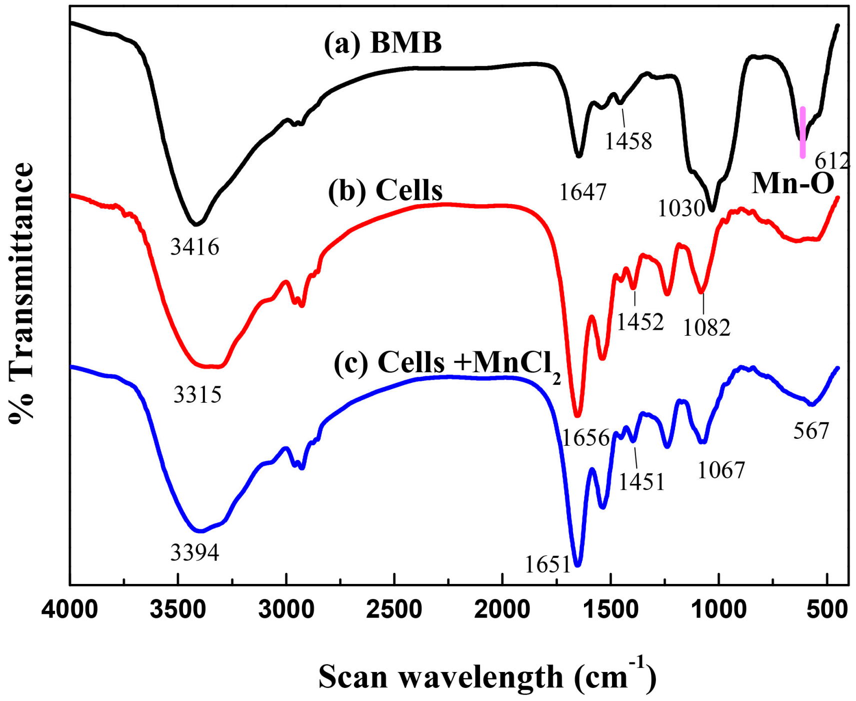

Figure S2. FT-IR spectra of the BMB, pure bacteria cells and pure bacteria cells with $MnCl_2$ without cultivation.

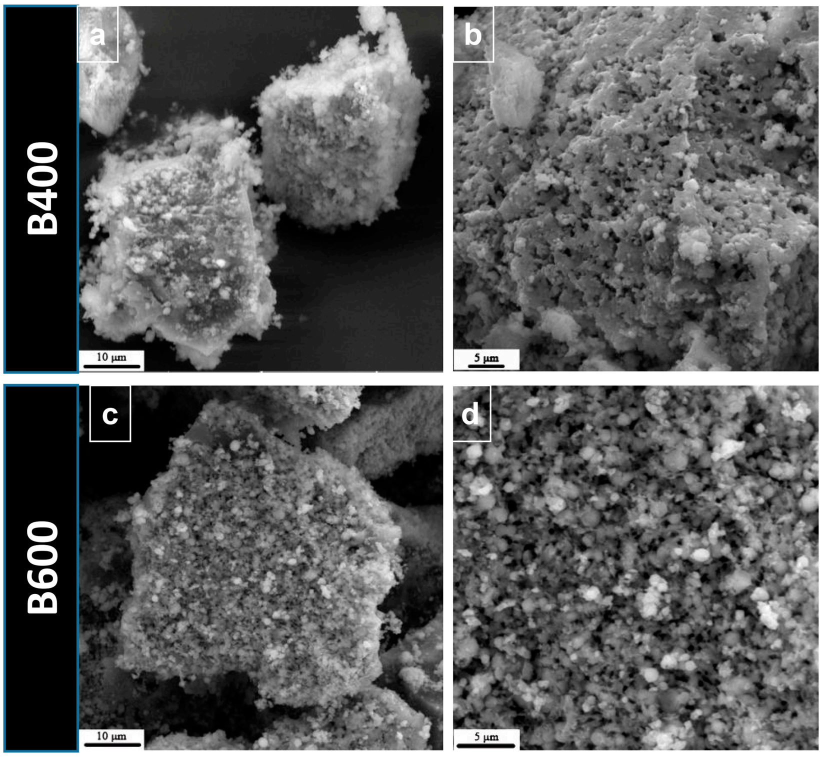

Figure S3. SEM images of the products prepared at (**a**, **b**) 400 °C (B400) and (**c**, **d**) 600 °C (B600).

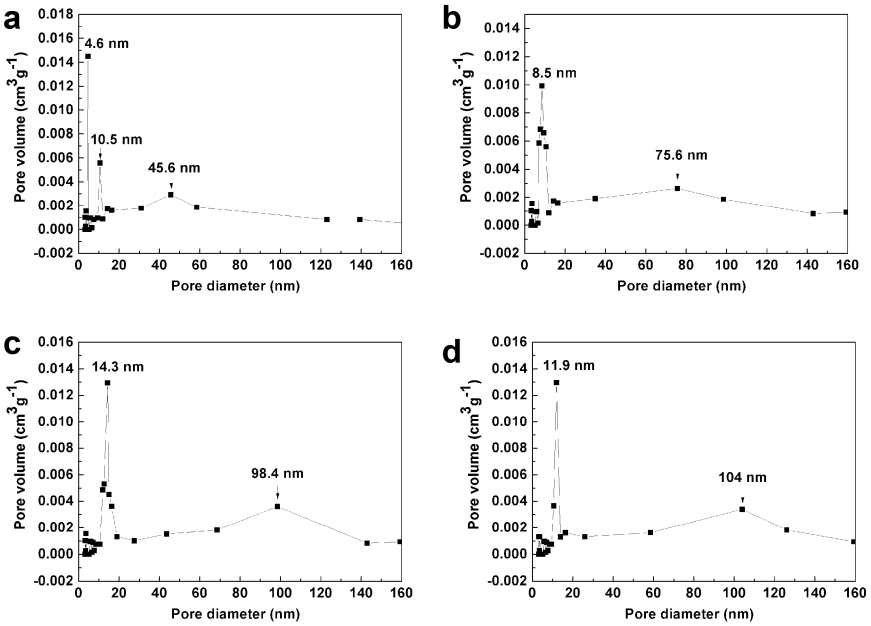

Figure S4. Pore size distribution curve of the samples under calcination conditions of (**a**) 400 °C (B400), (**b**) 600 °C (B600) and 800 °C (**c**: B800; **d**: CMB-Ni).

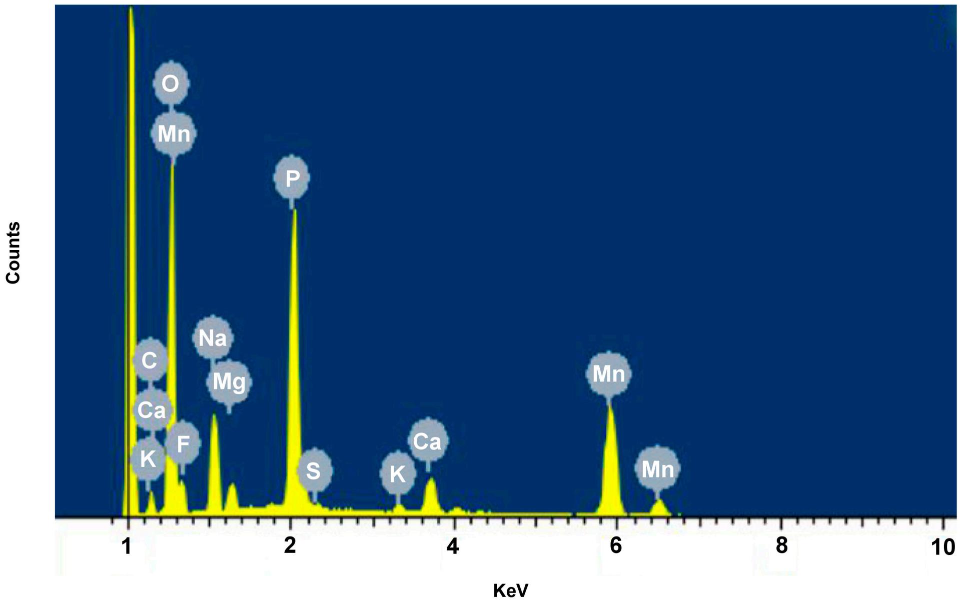

Figure S5. Line-scan SEM-EDS analysis of B800.

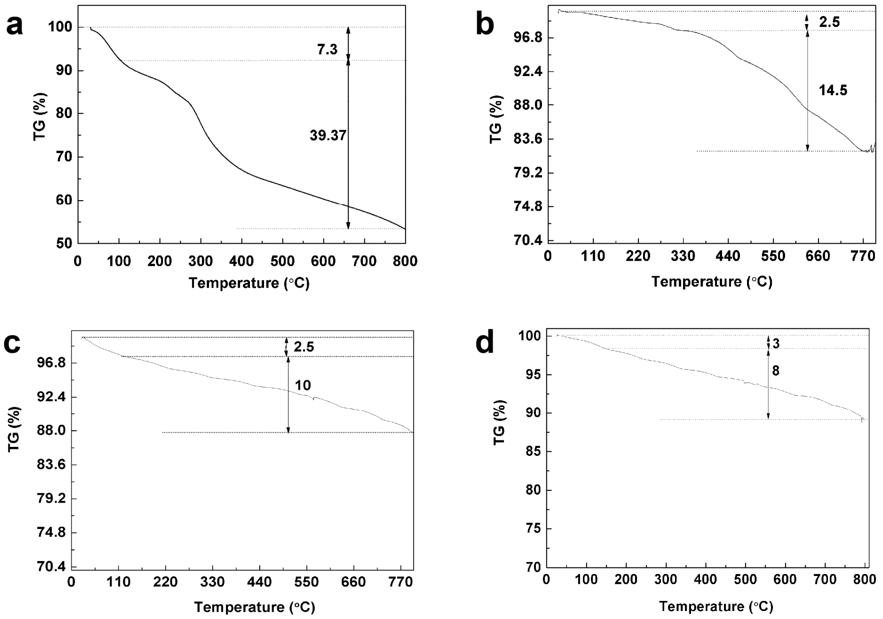

Figure S6. TGA-DSC curves of (**a**) B400, (**b**) B600, (**c**) B800 and (**d**) CMB-Ni.

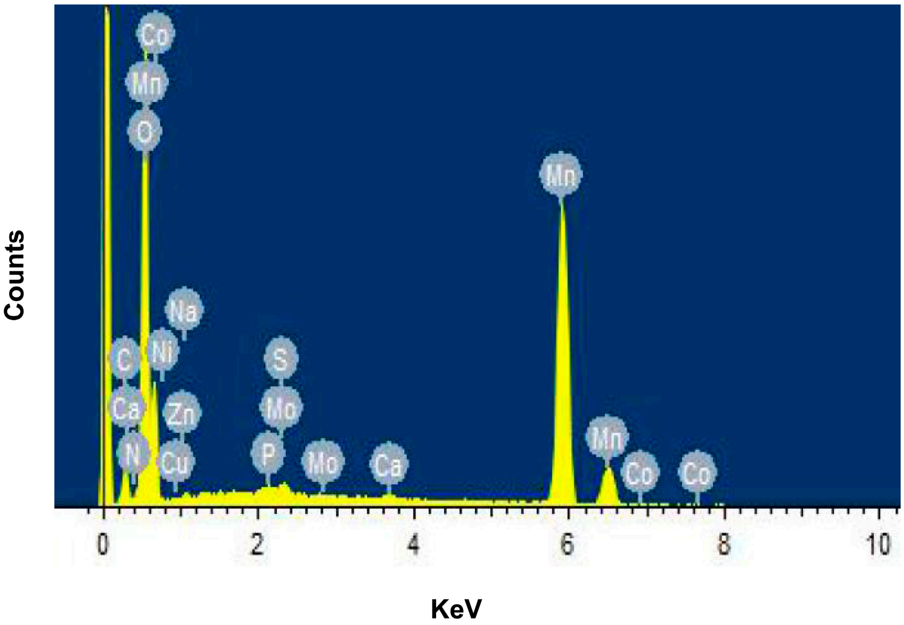

Figure S7. Line-scan SEM-EDS analysis of CMB-Ni.

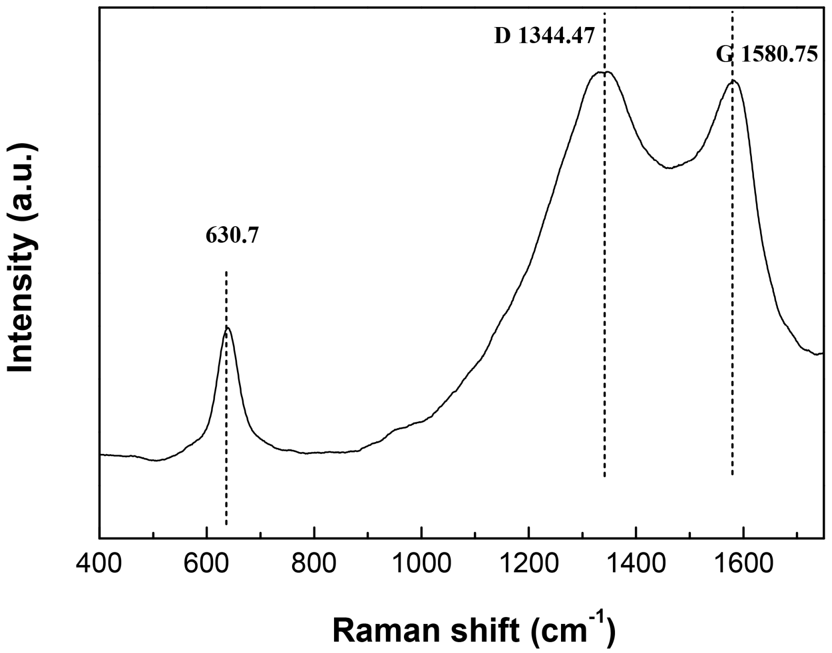

Figure S8. Raman spectra of B800 annealed under Ar atmospheres.

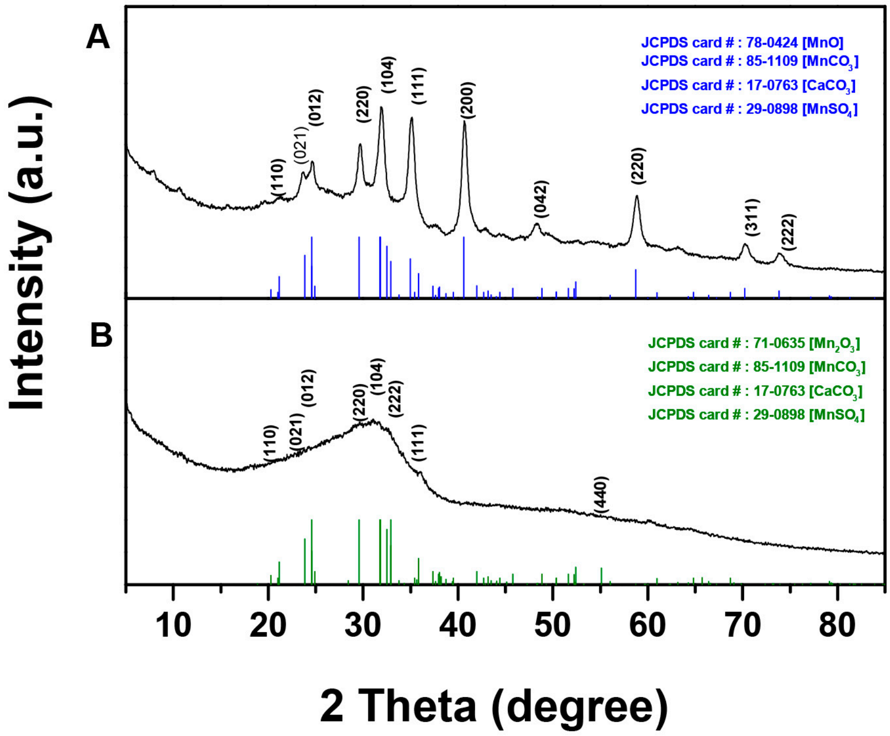

Figure S9. XRD patterns of (**A**) B600 and (**B**) B400. The Bragg positions and intensities are marked.

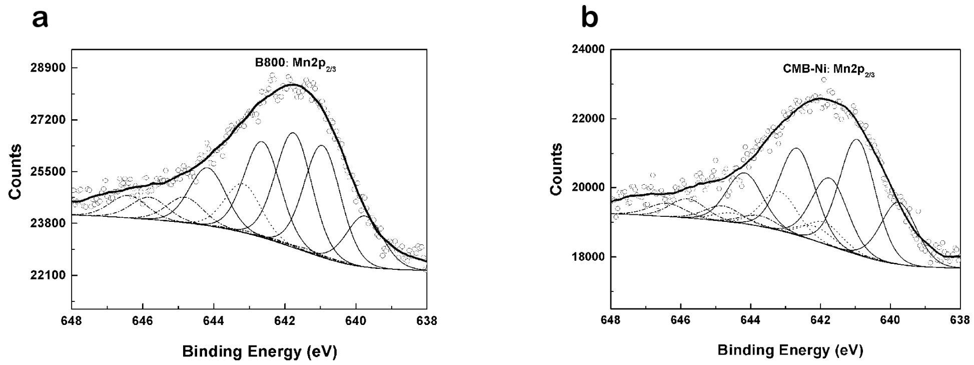

Figure S10. XPS patterns of the Mn (2p3/2) spectrogram of B800 (**a**) and CMB-Ni (**b**) prepared at 800 °C. The upper circles represent the observed data. The thick, solid curve indicates the best fit of the data. The dashed–dotted curves represent the Mn^{4+} multiplet peaks, the dotted lines represent Mn^{3+}, and the thin solid lines represent Mn^{2+}.
